# Pancreatic head clockwise devascularization technique during robotic pancreaticoduodenectomy to minimize intraoperative bleeding

**DOI:** 10.1007/s00464-025-12070-z

**Published:** 2025-08-28

**Authors:** Kyoji Ito, Yoshikuni Kawaguchi, Satoru Abe, Yuichiro Mihara, Yujiro Nishioka, Akihiko Ichida, Takeshi Takamoto, Nobuhisa Akamatsu, Kiyoshi Hasegawa

**Affiliations:** Hepato-Biliary-Pancreatic Surgery Division, Department of Surgery, Graduate School of Medicine, The University of Tokyo, -3-1 Hongo, Bunkyo-Ku, Tokyo, 113-8655 Japan

**Keywords:** Robotic pancreaticoduodenectomy, Artery-first approach, Novel technique

## Abstract

**Background:**

Pancreaticoduodenectomy (PD) is a complex procedure, and robotic PD (RPD) has been reported to have benefits in reducing postoperative complications. However, the timing and order for dividing arteries and veins remain unstandardized. We developed a novel technique, pancreatic head clockwise devascularization, to minimize intraoperative bleeding in RPD.

**Methods:**

We retrospectively analyzed 39 patients who underwent RPD between April 2022 and September 2024. The clockwise devascularization technique divides 1) the gastroduodenal artery, 2) the 1st-jejunal vein branches, and 3) the inferior pancreatoduodenal artery + 1st-jejunal artery. Outcomes were compared with the conventional superior mesenteric artery (SMA)-first approach.

**Results:**

Of the 39 patients, 14 were in the clockwise devascularization group and 25 in the SMA-first group. The clockwise group had a significantly shorter operation time (616 vs. 772 min, *P* < 0.01) and lower blood loss (50 vs. 330 ml, *P* = 0.03). There were no clinically relevant pancreatic fistulas or delayed gastric emptying in either group. The median hospital stay was shorter in the clockwise group (5.5 vs. 8.0 days, *P* < 0.01).

**Conclusions:**

The pancreatic head clockwise devascularization technique may be an effective technique to systematically devascularize the pancreatic head.

**Supplementary Information:**

The online version contains supplementary material available at 10.1007/s00464-025-12070-z.

Pancreaticoduodenectomy (PD) is a surgical procedure to treat diseases of the pancreatic head and duodenum that requires detailed anatomical understanding and technical proficiency [1, 2]. Robotic pancreaticoduodenectomy (RPD) has emerged as a promising alternative to conventional open surgery, offering improved visualization, dexterity, and precision in complex anatomical reconstructions [3–5]. The potential benefits of robotic surgery in reducing complications such as postoperative pancreatic fistula (POPF) and delayed gastric emptying (DGE) have generated interest among surgeons [6–8].

An artery-first approach is considered useful for minimizing bleeding and judging the resectability of pancreatic ductal adenocarcinoma by first dividing the arteries of the pancreatic head [9–12]. The artery-first approach for PD focuses on dissection around the superior mesenteric artery (SMA) and division of the inferior pancreaticoduodenal artery (IPDA) as the first step [13–16]. In addition to the IPDA, the gastroduodenal artery (GDA) is the main blood supply route to the pancreatic head. However, the artery-first approach does not focus on the timing of the GDA division. For patients with advanced pancreatic ductal adenocarcinoma of the pancreatic head, the artery-first approach (i.e., the SMA-first approach) is used to determine resectability in advance, followed by division of the GDA. However, this concept is not reasonable to patients undergoing PD for other indications. The timing and order to divide arteries and veins surrounding the pancreatic head have not been standardized yet. Therefore, we developed a systematic pancreatic head devascularization technique to minimize intraoperative bleeding, especially in RPD (termed the pancreatic head clockwise devascularization technique).

In this study, we present the details of the pancreatic head clockwise devascularization technique and compare the outcomes between patients who underwent this technique and those who did not.

## Methods

### Patients

Using a prospectively maintained database, we identified patients who underwent RPD at The University of Tokyo between April 2022 and September 2024. All surgeries were performed after obtaining informed consent from each patient. This study was approved by the Institutional Review Board of The University of Tokyo (no. 2158-11).

### Pancreatic head clockwise devascularization technique

**Fig. 1:**
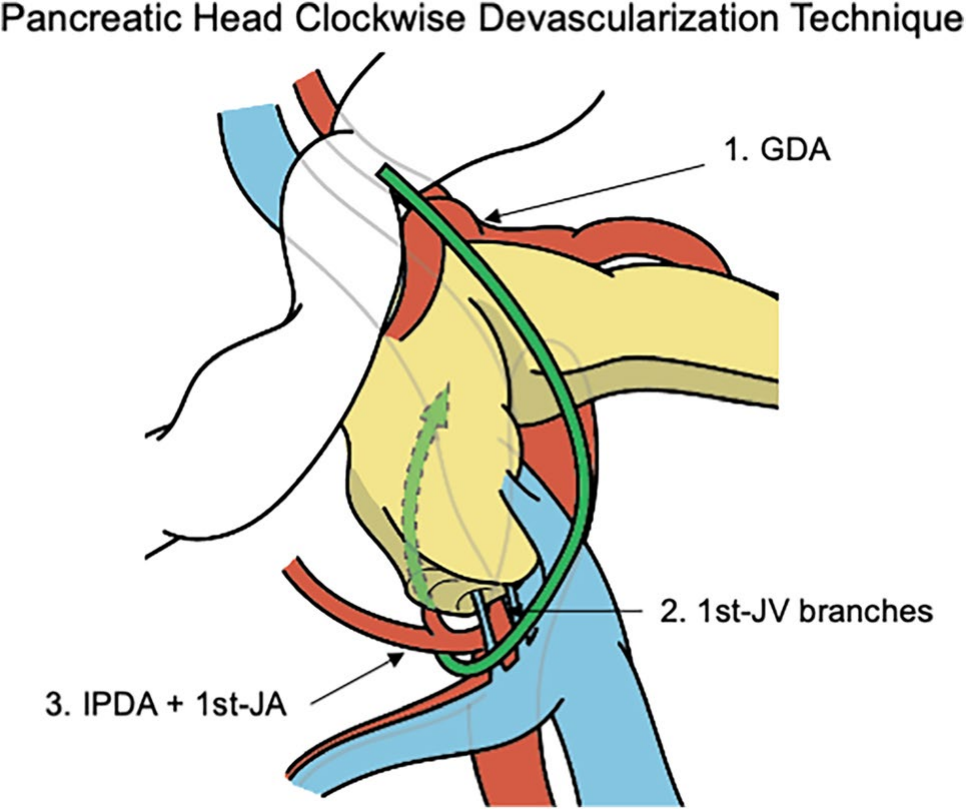
Scheme of pancreatic head clockwise devascularization technique. This technique divides 1) the GDA first, 2) the 1st-JV branches second, and 3) the IPDA + 1st-JA last. *GDA,* gastroduodenal artery; *IPDA*, inferior pancreatoduodenal artery; *JA*, jejunal artery; *JV*, jejunal vein

Patients were placed in a spread-leg supine position (head-up, 10°). Our standardized trocar placement, a 5-cm single umbilical incision with 2 ports, was used [8]. We originally performed the SMA-first approach that employed dissection and division of the IPDA + the first jejunal artery (1st-JA) using the right lateral approach [17], the first jejunal vein (1st-JV) branches including the inferior pancreatic dorsal vein (IPDV) from the pancreatic head (or the 1st-JV itself) followed by dissection and division of GDA: in other words, the order of division is 1) IPDA + 1st-JA, 2) 1st-JV branches, and 3) GDA. However, we occasionally encountered venous hemorrhage during dissection around the SMA. Therefore, for patients who do not need resectability determination around the SMA, we modified our technique to systematically devascularize the pancreatic head (termed the pancreatic head clockwise devascularization technique) (Fig. 1 and Supplementary Video 1) in the following three steps: division of 1) the GDA, 2) 1st-JV branches including the IPDV from the pancreatic head (or 1st-JV itself), and 3) IPDA + 1st-JA, with three different surgical views.

### Step 1: Division of the GDA with the in situ view

**Fig. 2:**
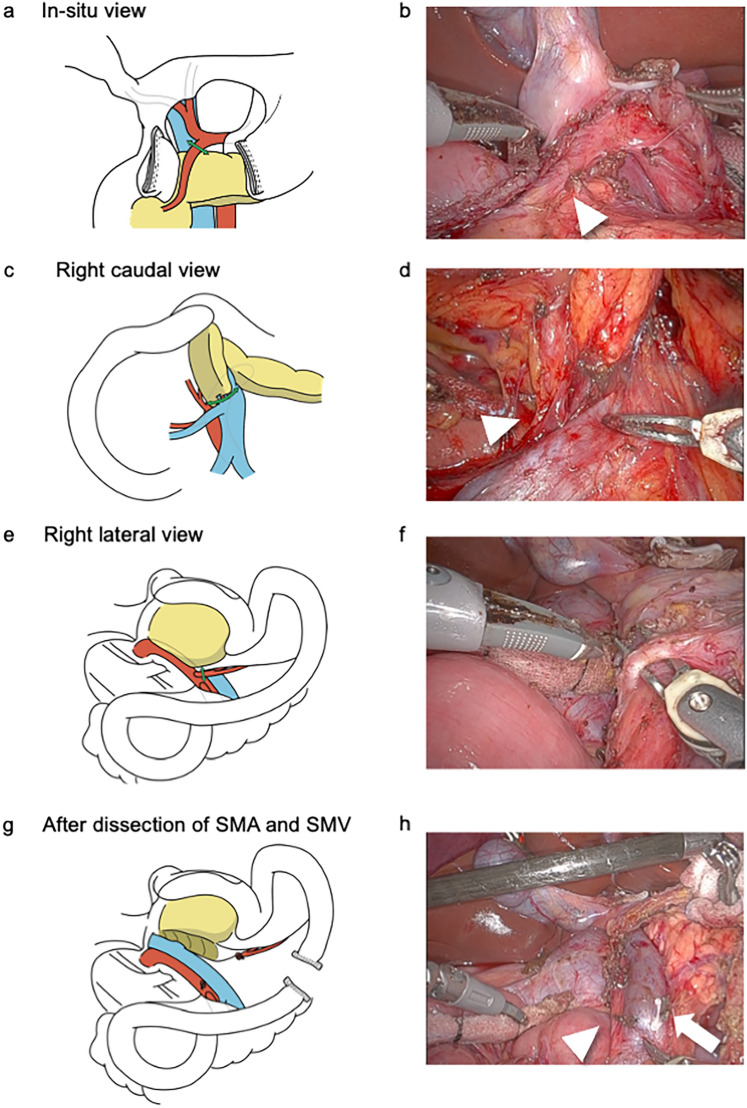
Details of pancreatic head clockwise devascularization technique. Illustrations (**a**, **c**, **e**, and **g**) and corresponding intraoperative gross appearances (**b**, **d**, **f**, and **h**). The GDA was divided after the GDA clamp test confirming no decrease of intrahepatic blood flow. Corresponding intraoperative gross appearances (arrowhead, GDA). **c** The 1st-JV branches were dissected from the pancreatic head and the 1st-JV was generally preserved. **d** Corresponding intraoperative gross appearances (arrowhead, the 1st-JV branches). **e** The right lateral wall of the SMA was dissected and exposed for its entire length to the cephalad, and the PL ph II was dissected along the SMA. **f** Corresponding intraoperative gross appearances. The IPDA + 1st-JA was encircled. **g** The SMV was exposed to the level of the SMA root, and the detachment of the pancreatic head from the SMA and SMV was completed with the right lateral view. **h** Corresponding intraoperative gross appearances (arrowhead, the SMA; arrow, the SMV). *GDA* gastroduodenal artery, *JA* jejunal artery, *JV* jejunal vein, *PL* pancreatic head plexus; *SMA* superior mesenteric artery, *SMV* superior mesenteric vein

The greater and lesser omentam were incised and the right gastric and epiploic arteries and veins were dissected peripherally. The stomach and duodenum were separated using a linear stapler. The GDA was identified and taped by dissecting the dorsal surface of the first portion of the duodenum and anterior surface of the pancreas. The GDA was dissected cephalad to identify the proper and common hepatic arteries. The GDA was divided after the GDA clamp test, confirming that there was no decrease in intrahepatic blood flow (Fig. 2a and b).

### Step 2: Division of the 1st-JV branches with the right caudal view

After mobilization of the third and fourth portions of the duodenum and jejunum, the jejunum was pulled to the right (a partial intestinal derotation [17, 18]). After identifying the second jejunal artery using indocyanine green-fluorescence imaging (Supplementary Fig. 1), the mesojejunum was incised to the left along the second jejunal artery (2nd-JA), and the SMA, 1st-JV, and superior mesenteric vein (SMV) were identified. The pancreatic head was retracted toward the left side and cephalad with the 3rd arm to maintain the pancreatic head in an upright position (right caudal view). The 1st-JV branches were dissected from the pancreatic head and the 1st-JV was generally preserved [19] (Fig. 2c and d). All venous branches from the inferior part of the pancreatic head (except Henle’s gastrocolic trunk) were divided and the 1st-JV was completely detached from the pancreatic head.

### Step 3: Division of IPDA + 1st-JA with the right posterior view

The pancreatic head was further rotated toward the left with the 3rd arm to form the right posterior view [20]. Detachment of the pancreatic head from the SMA and SMV was performed in the right posterior view with partial intestinal derotation The right lateral wall of the SMA was dissected and exposed cephalad for its entire length, and the pancreatic head nerve plexus II (PL ph II) was dissected along the SMA (Fig. 2e and f). The IPDA + 1st-JA were identified and divided. If the IPDA branched directly from the SMA, it was identified and divided. The SMV was exposed at the level of the SMA root, and detachment of the pancreatic head from the SMA and SMV was completed in the right posterior view (Fig. 2g and h). The jejunum was divided using a linear stapler.

After these steps were completed, the pancreatic head was placed in situ. After the division of the Henle’s gastrocolic trunk and IPDV, the pancreas was divided. Lymphadenectomy of the hepatoduodenal ligament was performed. The bile duct was divided, and the specimen was removed.

### Anatomical understanding of pancreatic head clockwise devascularization technique with detailed illustrations

**Fig. 3:**
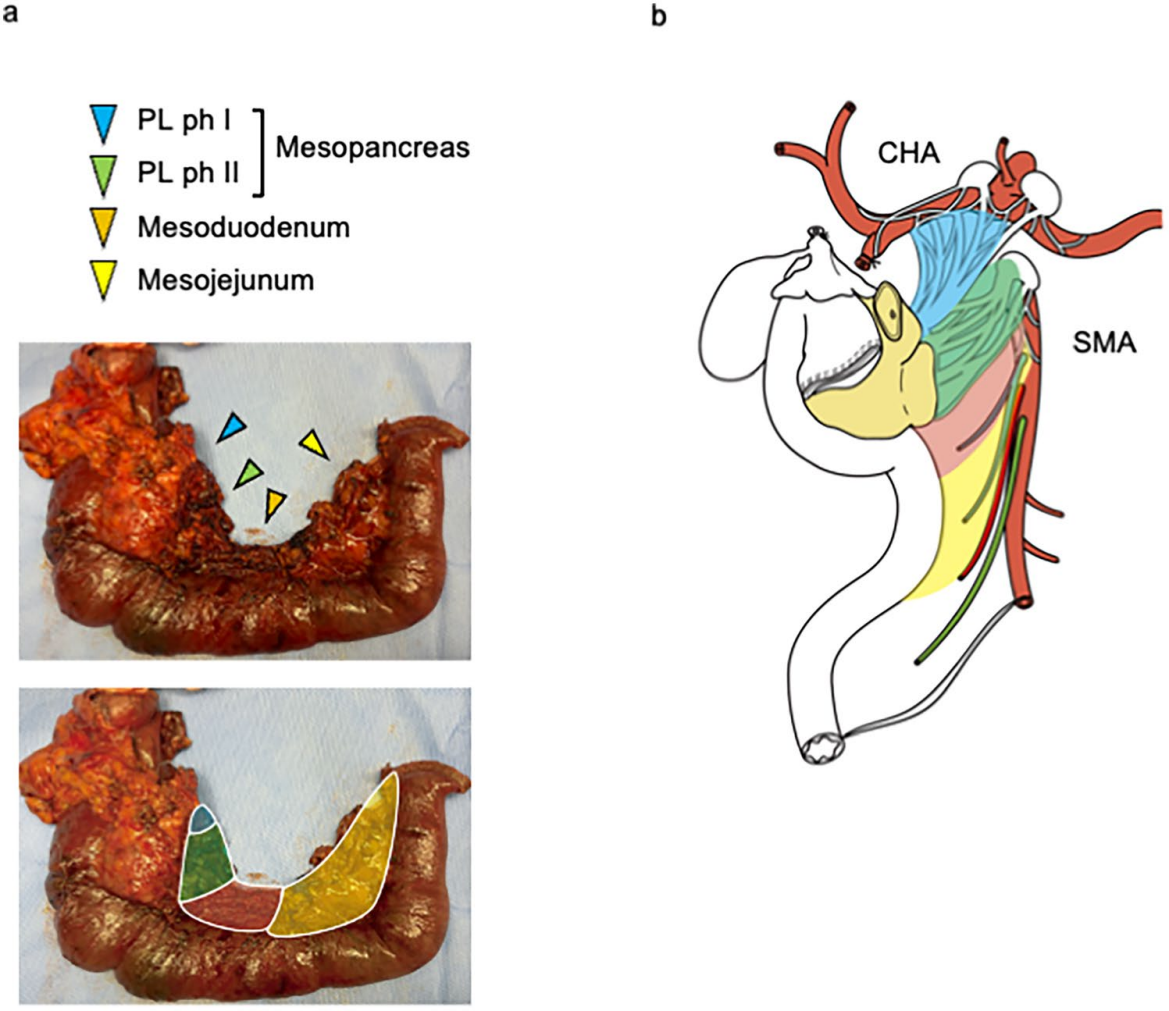
Mesopancreas, mesoduodenum, and mesojejunum. **a** Mesopancreas, mesoduodenum, and mesojejunum in the resected specimen. **b** Schema of mesopancreas, mesoduodenum, and mesojejunum

**Fig. 4:**
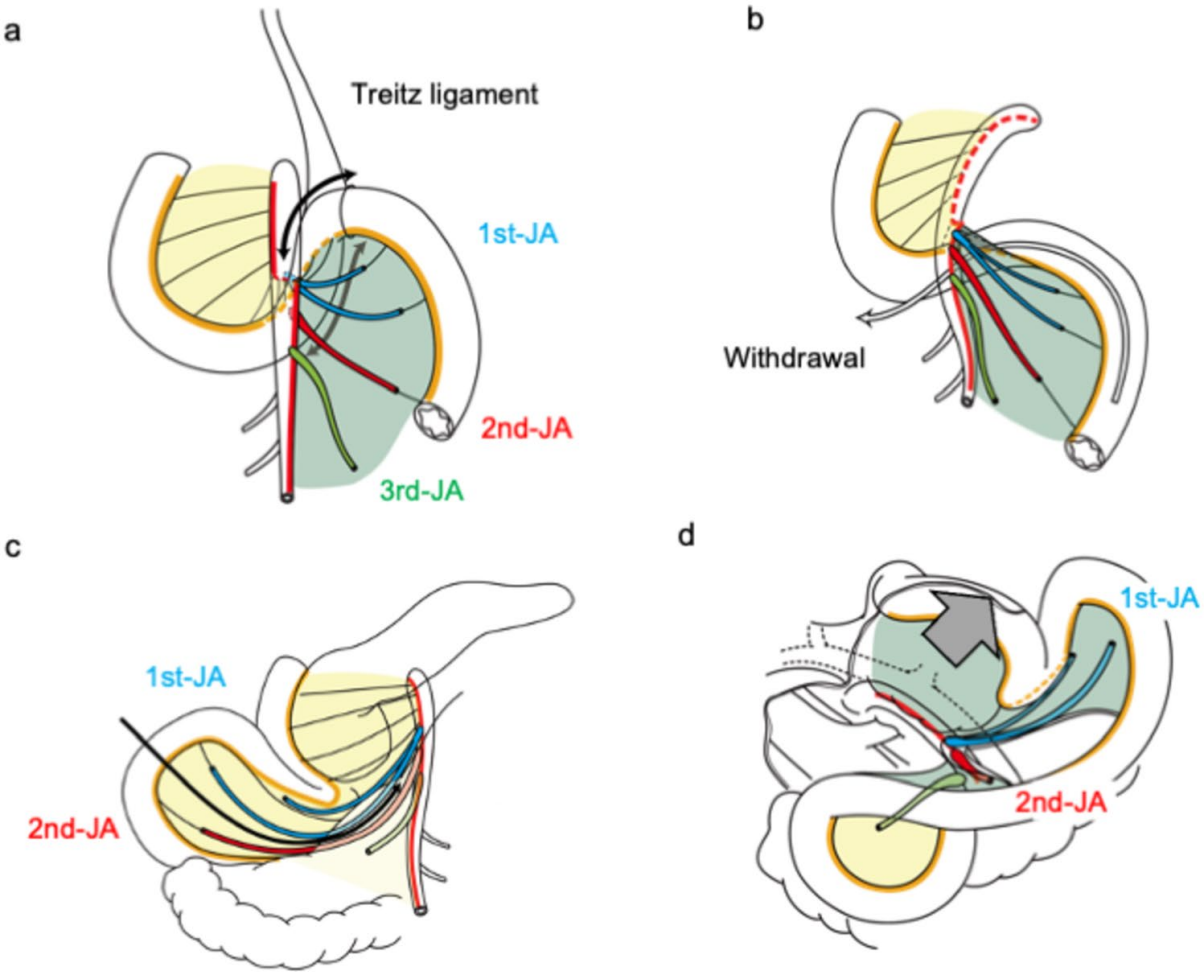
Anatomical understanding of pancreatic head clockwise devascularization technique. The ventral aspect of the mesentery after withdrawal is colored in yellow, and the opposite side is colored in green. **a** The ligament of Treitz is divided, and the third and fourth portions of the duodenum were dissected. **b** The third and fourth portions of the duodenum and jejunum (approximately 30 cm) were pulled to the right. **c** The mesojejunum was dissected along with the 2nd-JA. **d** The pancreatic head was retracted toward the left side and cephalad, and the PL ph II was dissected from the SMA in the right posterior view. *JA*, jejunal artery; *PL*, pancreatic head plexus; *SMA*, superior mesenteric artery

Figures 3 and 4 show detailed illustrations and pictures of resected specimens to understand anatomical details for pancreatic head clockwise devascularization technique.

1) Anatomy of the mesopancreas, mesoduodenum, and mesojejunum

Figure 3a shows the mesopancreas, mesoduodenum, and mesojejunum of the resected specimens with a corresponding illustration (Fig. 3b) [21, 22]. These mesenteries originate from the SMA; the mesopancreas is attached to the pancreas, the mesoduodenum to the duodenum, and the mesojejunum to the jejunum with internal nutrient vessels. The mesopancreas is divided into PL ph I and II [22].

2) Partial derotaion of the duodenum and jejunum

After cutting the ligament of Treitz (Fig. 4a), the third and fourth portions of the duodenum and jejunum (approximately 30 cm) were pulled to the right (Fig. 4b and c). This partial derotation technique differs from the original intestinal derotation technique, which mobilizes all the small intestine and right colon [23, 24] (Supplementary Fig. 2).

3) Dissection of the mesojejunum following the 2nd-JA

The mesojejunum was dissected along the 2nd-JA (Fig. 4c). The 1st-JV branches were dissected from the pancreatic head in the right caudal view.

4) Dissection of the PL ph II

The pancreatic head was retracted toward the left side and cephalad (Fig. 4d), and PL ph II was dissected from the SMA in the right posterior view.

### Techniques of pancreatojejunostomy and gastrojejunostomy

Pancreatojejunostomy was performed using a modified Blumgart anastomosis and was designed according to the shape of the stump of the pancreas and the location of the main pancreatic duct. Suturing and ligation were carefully performed to avoid re-suturing [25]. Gastrojejunostomy was performed in an open setting through the umbilical wound after undocking the robot [25]. The lengths of the afferent and efferent loops were adjusted to ensure that the stomach was positioned straight downward on the left side of the body after anastomosis. An anastomotic opening was created with a 60-mm linear stapler with staple line reinforcement in a side-to-side fashion.

### Postoperative management

We implemented our Enhanced Recovery After Surgery program, the Fast-Track Return to Normal Activity (FASTOL) program, in patients undergoing minimally invasive surgery [8]. Oral intake and mobilization exercises were initiated on postoperative day (POD) 1. Supportive peripheral parenteral nutrition targeted 20 kcal/kg and 0.8 g/kg of amino acid per day during PODs 1–4. The criteria for hospital discharge were as follows: no postoperative complications were observed, intravenous analgesia was not needed, and patients could perform activities of daily living.

### Definitions

The American Society of Anesthesiologists classification [26] was used to determine the patients’ preoperative physical fitness, which was recorded based on the preoperative assessment of the anesthesiologists. Clinically relevant postoperative pancreatic fistula (CR-POPF) was defined based on the 2016 updated International Study Group of Pancreatic Surgery definitions [27]. DGE was performed as described by the International Study Group for Pancreatic Surgery [28]. Surgical and postoperative outcomes were compared between patients who underwent the clockwise devascularization technique and those who underwent the conventional SMA-first approach.

### Statistical analysis

Categorical variables are expressed as numbers and percentages. Continuous variables were expressed as median values with interquartile ranges. Statistical analyses were performed using R version 4.3.1 (R Foundation for Statistical Computing, Vienna, Austria).

## Results

### Patients

Between January 2023 and September 2024, 39 consecutive patients underwent RPD. A single surgeon initiated the RPD in January 2023 and performed all RPD during the study period. Demographic and clinicopathological variables are shown in Table 1. The median patient age (interquartile range [IQR]) was 68.0 years (60.0–74.5). The two major indications for RPD were intraductal papillary mucinous neoplasms (19/39, 48.7%) and ampulla of Vater tumor (12/39, 30.8%). After the 24th patient, the pancreatic head clockwise devascularization technique was generally used (the clockwise devascularization group, *n* = 14), except for one patient who had an endoscopic nasobiliary drainage tube. The remaining patients underwent the conventional SMA-first approach (the SMA-first group, *n* = 25).

**Table 1:** Patient characteristics and surgical outcomes

Characteristics	All Patients *n* = 39	Clockwise devascularization group *n* = 14	SMA-first group *n* = 25	*P* value
Demographics and clinicopathological variables				
Age, median (IQR), yrs	68.0 (60.0–74.5)	69.0 (65.2–77.5)	68.0 (56.0–73.0)	0.16
Sex, male: female, *n*	17: 22	4: 10	13: 12	0.28
BMI, median (IQR), kg/m2	22.3 (19.4–24.9)	22.4 (20.2–24.4)	22.3 (19.3–24.9)	0.76
ASA score ≥ 3, *n* (%)^*§*^	7 (17.9)	2 (14.3)	5 (20.0)	0.99
Diagnosis, *n* (%)				0.20
IPMN	19 (48.7)	9 (64.3)	10 (40.0)	
Ampulla of Vater tumor	12 (30.8)	2 (14.3)	10 (40.0)	
NET	5 (12.9)	3 (21.4)	2 (8.0)	
Others	3 (7.7)	0	3 (12.0)	
Surgical and postoperative outcomes				
Operation time, median (IQR), min	683.0 (616.0–801.0)	616.0 (568.5–654.8)	772.0 (683.0–817.0)	< 0.01
Blood loss, median (IQR), ml	270.0 (50.0–540.0)	50.0 (17.5–255.0)	330.0 (120.0–600.0)	0.03
CR-POPF, *n* (%)	0	0	0	NA
DGE, *n* (%)	0	0	0	NA
Postoperative bleeding, *n* (%)	0	0	0	NA
Fluid collection, *n* (%)	4 (10.3)	0	4 (16.0)	0.30
Hospital stay, median (IQR), day	7.0 (6.0–9.0)	5.5 (5.0–6.0)	8.0 (7.0–9.0)	< 0.01
30-day readmission,* n* (%)	5 (12.8)	1 (7.1)	4 (16.0)	0.768
30-day reoperation, *n* (%)	0	0	0	NA
90-day mortality, *n* (%)	0	0	0	NA

*CR-POPF* clinically relevant postoperative pancreatic fistula, *DGE* delayed gastric emptying, *IQR* interquartile range, *IPDA* inferior pancreaticoduodenal artery, *IPMN* intraductal papillary mucinous neoplasm, *NET* neuroendocrine neoplasm

### Surgical and postoperative outcomes

The surgical and postoperative outcomes of the 39 patients who underwent RPD are shown in Table 1. The operation time was significantly shorter in the clockwise devascularization group than in the IPDA-first group (616 min [569–655] vs. 772 min [683–817], *P* < 0.01). The estimated blood loss was significantly lower in the clockwise devascularization group than in the IPDA-first group (50 ml [18–255] vs. 330 ml [120–600], *P* = 0.03). None of the patients in either group developed CR-POPF, DGE, or bile leakage. Fluid collection developed in 4 patients. One patient underwent radiographic drainage but the amylase level of the discharge was not elevated. The other three patients didn’t show sustained inflammation and the fluid collection was alleviated by diuretics. The median (IQR) length of hospital stay was significantly shorter in the clockwise devascularization group than in the IPDA-first group: 5.5 days (5.0–6.0) vs. 8.0 days (7.0–9.0), P < 0.01. None of the patients in either group experienced 30–day reoperation and 90–day mortality in both groups.

## Discussion

Our study detailed the pancreatic head clockwise devascularization technique during RPD. This technique consisted of the following three steps: division of 1) the GDA in the in situ view, 2) the 1st-JV branches in the right caudal view, and 3) the IPDA + 1st-JA in the right posterior view. Our preliminary findings showed that patients who underwent the pancreatic head clockwise devascularization technique had less blood loss and shorter operating times compared to those who underwent the conventional SMA-first approach. This technique is reasonable and effective for systematically devascularizing the pancreatic head during PD.

The artery-first approach for PD is performed by dividing the arteries into the pancreatic head before dividing the pancreatic head veins, and is reported to have the following four effects: (1) reduction of intraoperative blood loss, (2) early determination of arterial involvement by the tumor, (3) clearance of surgical margins along the arteries, and (4) ultimately, a more oncologically complete resection [9–11]. The artery-first approach for PD is generally regarded as a dissection around the SMA and division of the IPDA in the first step [13–16]. However, the GDA also provides arterial blood flow to the pancreatic head. Gundara et al. reported a strategy to reduce hemorrhage in PD by dividing not only the IPDA but also the GDA prior to division of the pancreatic head veins to achieve early and complete pancreatic head devascularization [29]. However, the artery-first approach does not focus on the timing of the GDA division. Our technique divides 1) the GDA first, 2) the 1st-JV branches second, and 3) the IPDA + 1st-JA last if resectability determination around the SMA is not necessary. Our technique is based on the following three reasonable steps. First, we divide the GDA to reduce the blood supply to the pancreatic head. Second, we prioritize the division of the 1st-JV branches using the right caudal view before dividing the IPDA + 1st-JA. That is because we encountered bleeding from the 1st-JV branches (the small drainage veins from the pancreatic head to the 1st-JV) when dissecting the IPDA + 1st-JA in the SMA-first approach. Indeed, previous studies reported that the1st-JV caused unintended excessive bleeding during PD [30–32]. The right caudal view is particularly effective for safely dividing the 1st-JV branches, because the thin veins from the pancreatic head to the 1st-JV are clearly visualized from the anterior perspective. When dividing the 1st-JV branches, we preserve Henle’s gastrocolic trunk to avoid the congestion of the pancreatic head. Finally, we safely divide the IPDA + 1st-JA with the reduced blood supply to the pancreatic head and no surrounding veins that cause bleeding. As such, we do not term our technique the artery-first approach but rather the pancreatic head clockwise devascularization technique, which divides 1) the GDA, 2) the 1st-JV branches, and 3) the IPDA + 1st-JA. This technique is especially useful for RPD because the caudal and right posterior views are facilitated by laparoscopy.

The landscape of minimally invasive pancreaticoduodenectomy is continuously evolving, with ongoing debates regarding the optimal approach between robotic and laparoscopic platforms. RPD offers distinct advantages such as enhanced 3D visualization, improved dexterity, and tremor filtration, which are particularly beneficial for complex dissections and anastomoses [33]. Conversely, laparoscopic PD may present challenges in certain complicated maneuvers due to limitations in instrument articulation [33]. Meta-analyses suggested that RPD may offer advantages in terms of lower conversion rates to open surgery [34, 35], reduced blood loss [35], reduced transfusion requirements [34], shorter hospital stay [34], and the lower mortality [36].

Our pancreatic head clockwise devascularization technique, with its emphasis on specific surgical views (caudal and right posterior views) and precise vessel division, is particularly well-suited for the robotic platform. The magnified and stable field of view provided by robotic systems facilitates the intricate vascular dissection described in our technique. This highlights how our approach utilizes the unique capabilities of robotic surgery to potentially enhance surgical safety and efficiency. It is important to acknowledge that a zero incidence of CR-POPF was achieved in the series. We covered the pancreatojejunostomy with an absorbable reinforcement felt made of polyglycolic acid and sprayed a fibrin sealant [25]. To reduce the risk of pancreatic fistula, studies reported techniques to wrap the GDA stump with the ligamentum teres and pancreatojejunostomy with the omentum flap [37, 38].

Our study should be understood in the context of the following limitations. First, this is a retrospective report on the experience of a single surgeon at a single institution. Second, the sample size of our study was relatively small (39 patients). A larger cohort would provide a more robust assessment of the efficacy of our approach across different patient demographics and disease presentations. Finally, it is unknown whether the improvement in operative time and blood loss in patients undergoing the pancreatic head clockwise devascularization technique should be attributable to this method or the learning curve because our data are preliminary and based on the initial consecutive cases of a single surgeon.

## Conclusions

Our study highlights the effectiveness of the systematic pancreatic head devascularization technique during RPD. The pancreatic head clockwise devascularization technique was associated with lower blood loss, and may be a reasonable and effective technique to systemically devascularize the pancreatic head during PD.

## Supplementary Information

Below is the link to the electronic supplementary material.

Supplementary file1 (DOCX 3931 KB)

## Abbreviations

CR-POPF: Clinically relevant postoperative pancreatic fistula
DGE: Delayed gastric emptying
FASTOL: Fast-track return to normal activity
GDA: Gastroduodenal artery
IQR: Interquartile range
IPDA: Inferior pancreaticoduodenal artery
IPDV: Inferior pancreaticoduodenal vein
JA: Jejunal artery
JV: Jejunal vein
PD: Pancreaticoduodenectomy
PL ph: Pancreatic head nerve plexus
POPF: Postoperative pancreatic fistula
RPD: Robotic pancreaticoduodenectomy
SMA: Superior mesenteric artery
SMV: Superior mesenteric vein
