# Supplementary material for: Pancreatic head clockwise devascularization technique during robotic pancreaticoduodenectomy to minimize intraoperative bleeding
Source: Surg Endosc. 2025 Aug 28;39(11):7347–55. doi: 10.1007/s00464-025-12070-z (PMC12618361; doi:10.1007/s00464-025-12070-z)

**Supplementary Figure Legends**

**Supplementary Figure 1. Identification of the first (arrowhead) and second jejunal artery (arrow) using indocyanine green-fluoresce imaging.**

**Supplementary Figure 2. Original intestinal derotation technique which mobilizes all the small intestine and the right colon.**

**Supplementary Video Legends**

**Supplementary Video 1. Pancreatic head devascularization technique**

**Step 1: Division of the gastroduodenal artery (GDA) with the in-situ view**

After division of the stomach using a linear stapler, the GDA was identified and taped by dissecting the dorsal surface of the first portion of the duodenum and anterior surface of the pancreas. The GDA was dissected cephalad to identify the proper and common hepatic arteries. The GDA was divided after the GDA clamp test, confirming that there was no decrease in intrahepatic blood flow.

After Kocherization and mobilization of the third and fourth portions of the duodenum and jejunum, the jejunum was pulled to the right. After identifying the second jejunal artery using indocyanine green-fluorescence imaging, the mesojejunum was incised to the left along the second jejunal artery, and the superior mesenteric artery (SMA), first jejunal vein (1st-JV), and superior mesenteric vein (SMV) were identified.

**Step 2: Division of the 1st-JV branches with the right caudal view**

The pancreatic head was retracted toward the left side and cephalad with the 3rd arm to maintain the pancreatic head in an upright position (right caudal view). The 1st-JV branches were dissected from the pancreatic head. All venous branches from the inferior part of the pancreatic head (except Henle's gastrocolic trunk) were divided and the 1st-JV was completely detached from the pancreatic head.

**Step 3: Division of** inferior pancreaticoduodenal artery (**IPDA) + first jejunal artery (1st-JA) with the right posterior view**

The pancreatic head was further rotated toward the left with the 3rd arm to form the right posterior view. Detachment of the pancreatic head from the SMA and SMV was performed in the right posterior view with partial intestinal derotation. The right lateral wall of the SMA was dissected and exposed cephalad for its entire length, and the pancreatic head nerve plexus II was dissected along the SMA. The IPDA + 1st-JA were identified and divided. The SMV was exposed at the level of the SMA root, and the Henle's gastrocolic trunk was divided. The detachment of the pancreatic head from the SMA and SMV was completed in the right posterior view.


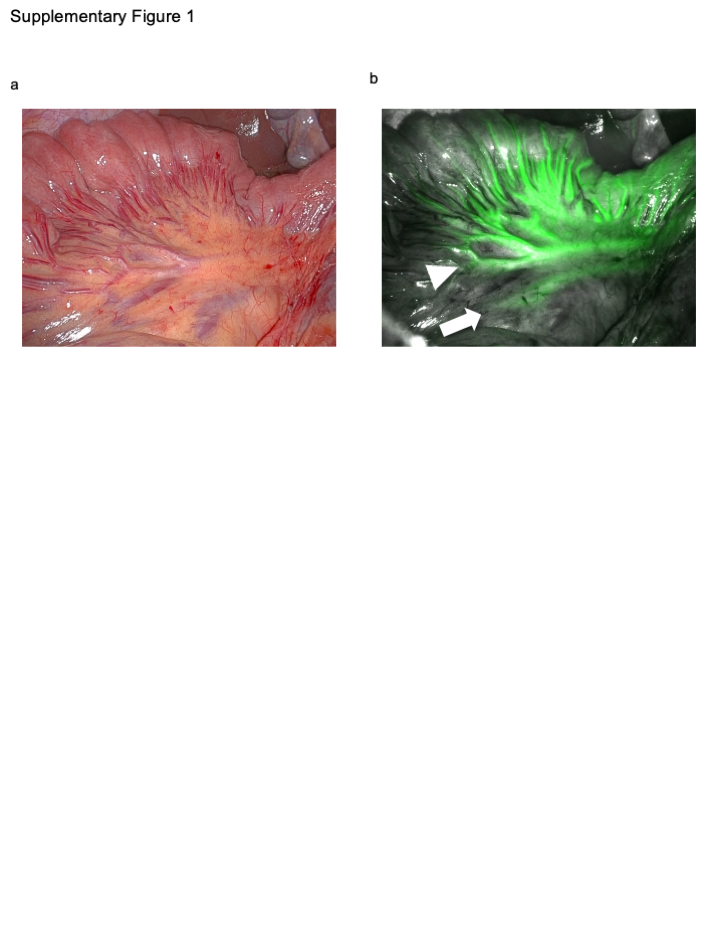


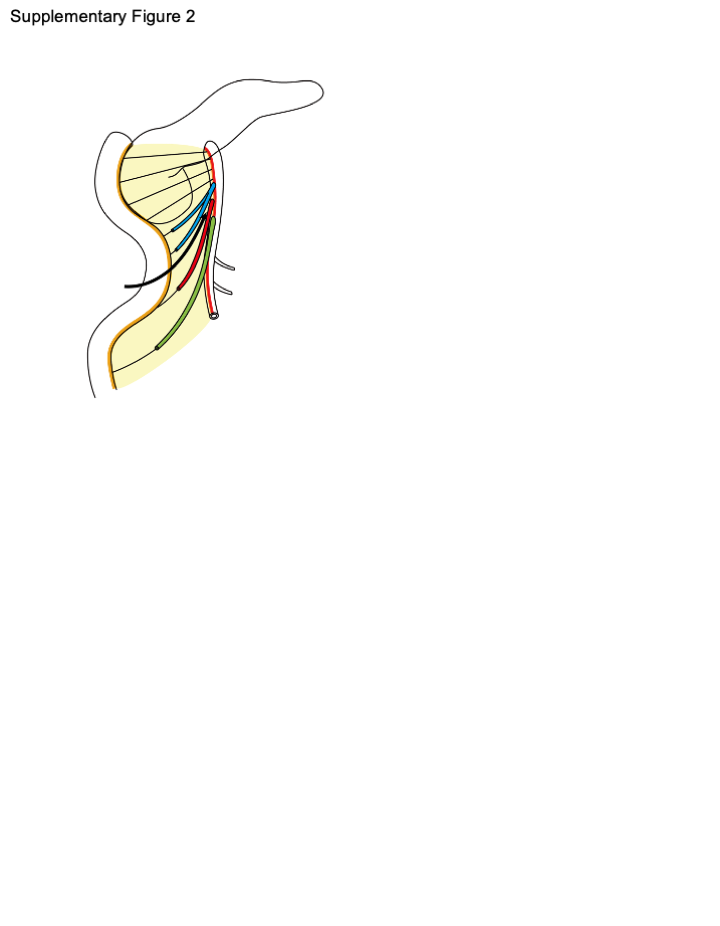

Supplement: Supplementary file 1 — Supplementary file1 (DOCX 3931 KB) [file 464_2025_12070_MOESM1_ESM.docx]
